# Using Community-Based Participatory Research Strategies to Promote Liver Cancer Prevention

**DOI:** 10.3390/socsci14110639

**Published:** 2025-10-31

**Authors:** Lin Zhu, Wenyue Lu, Ming-Chin Yeh, Grace X. Ma, Evelyn T. González, Kerry Traub, Marilyn A. Fraser, Nathaly Rubio-Torio, Ada Wong, Yin Tan

**Affiliations:** 1 Center for Asian Health, Lewis Katz School of Medicine, Temple University, 3440 N Broad Street, Philadelphia, PA 19140, USA;; 2 Department of Urban Health and Population Science, Lewis Katz School of Medicine, Temple University, 3440 N Broad Street, Philadelphia, PA 19140, USA; 3 Department of Nutrition and Public Health, Hunter College, City University of New York, 2180 Third Ave., New York, NY 10035, USA;; 4 Office of Community Outreach, Fox Chase Cancer Center, Temple University Health System, 333 Cottman Ave., Philadelphia, PA 19111, USA;; 5 Arthur Ashe Institute for Urban Health, 450 Clarkson Ave. # 1232, Brooklyn, New York, NY 11203, USA;; 6 Voces Latinas Inc., 37-63C 83rd St 2nd Floor, Jackson Heights, New York, NY 11372, USA;; 7 New York City Council, District 43, City Hall Park, New York, NY 10007, USA;

**Keywords:** community-based participatory research, community health, capacity building, health promotion, health disparities

## Abstract

Hispanic, Asian, and African Americans are disproportionately affected by liver cancer, viral hepatitis B (HBV), and viral hepatitis C (HCV), in part because of barriers to liver cancer awareness and prevention. We implemented a community-based, culturally tailored initiative to raise awareness and promote uptake of behaviors for liver cancer prevention, early diagnosis, monitoring, and treatment. Guided by community-based participatory research (CBPR) principles and built on well-established collaboration with community-based organizations, we actively engaged the community advisory board (CAB), community health workers, and community members in multiple phases of (1) a community-based educational initiative, (2) a city-wide bus campaign, and (3) community health fairs. This multilevel initiative saw notable changes in community members’ knowledge of liver cancer, viral hepatitis, lifestyle behaviors like dietary patterns, and uptake of screening tests for HBV/HCV. Additionally, the comprehensive engagement of CAB, healthcare workers, and community members significantly benefited community capacity building on cancer research and health promotion. These CBPR-guided community initiatives had remarkable positive impacts on promoting liver cancer awareness and prevention among underserved racial/ethnic minorities. The academic–community relationships built on and strengthened through shared power, mutual respect, and trust serve as the foundation for sustainable community growth and empowerment.

## 1. Introduction

Liver cancer incidence rates have more than tripled since 1980, making it one of the most commonly diagnosed and deadliest cancers in the United States (Salvatore et al. 2019; Siegel et al. 2021). Hispanic, Asian, and African Americans, in particular, experience an overall greater burden of the disease (Siegel et al. 2021). Chronic infections with hepatitis B virus (HBV) and hepatitis C virus (HCV) are considered the most common risk factors of liver cancer, causing 80% of hepatocellular carcinoma cases (Kim and El-Serag 2019) and 65% of liver cancer cases (Siegel et al. 2021). Hispanic, Asian, and African Americans are disproportionately impacted by HBV (Hu et al. 2011; Estrada 2005) and HCV infection (Estrada 2005; Gordon et al. 2019). Research has shown that these three populations experience significant barriers to liver cancer prevention information, prevention resources, and healthcare services concerning viral hepatitis and liver cancer (Tatar et al. 2020; Fang and Stewart 2018). Such barriers exist on multiple levels: individual, interpersonal, community, sociocultural, healthcare system, and structural (Fang and Stewart 2018).

Community-based participatory research (CBPR) has been established as an effective way of raising awareness of HBV/HCV and liver cancer screening, prevention, and treatment (Ma et al. 2018, 2023). The CBPR approach is defined as a collaborative partnership in which researchers, community members, and organizational representatives share power, mutual trust, and recognition in every aspect of the research process and outcome, including intervention strategy determination and development, research question and measurement development, data collection, interpretation, and dissemination of findings (Israel et al. 1998). One of the key principles of CBPR is to facilitate a collaborative and equitable partnership in all stages of research. CBPR is an effective approach to reducing health disparities through involving underserved communities and enhancing the effectiveness of intervention strategies (Ma et al. 2023; Zhu et al. 2022).

Guided by the CBPR approach, large-scale clustered randomized trials conducted among underserved Korean and Vietnamese American participants showed that intervention strategies adherent to CBPR principles effectively enhanced HBV screening and vaccination among underserved Korean and Vietnamese Americans (Ma et al. 2018). Among African Americans, engaging and educating community members to deliver community-based interventions is promising in reducing HCV infection (Kempf et al. 2018). The application of a CBPR approach to better understand hepatitis screening and vaccination among Hispanic Americans is limited. Nonetheless, the CBPR approach has proven to be successful in recruiting Hispanic (Thompson et al. 2010) and African American (McNeill et al. 2018) participants for cancer prevention research.

The goals of this study are to describe the use of CBPR principles in cancer prevention partnership outreach initiatives and to investigate how these methods impacted the planning and implementation of multilevel community initiatives on cancer presentation through engagement with the community to address cancer disparities in low-income, underserved minority communities. In addition, we report successes and lessons learned in implementing the community initiatives, including the challenges brought or exacerbated by the COVID-19 pandemic.

## 2. Materials and Methods

The Synergistic Partnership for Enhancing Excellence in Cancer Health (SPEECH) is a comprehensive regional cancer health disparity partnership between Temple University/Fox Chase Cancer Center (TUFCCC) and Hunter College (HC) that is funded by a U54 grant from the National Cancer Institute. The purpose of SPEECH is to reduce cancer health disparities among underserved minority populations in the Pennsylvania-New Jersey-New York City (PNN) region through cancer disparities research, community outreach, and career development for underrepresented early-stage investigators and students. A critical component of the SPEECH partnership is the Community Outreach Core (COC). One of the aims of COC is to increase cancer prevention awareness and improve behaviors related to cancer prevention and early diagnosis through three community-wide outreach initiatives in African American, Asian American, and Hispanic communities in the PNN region. Each initiative was designed to last for 24 months, but the implementation period was impacted by the COVID-19 pandemic. The first initiative is focused on liver cancer and viral hepatitis (from October 2018 to March 2021), the second on colorectal cancer (from April 2021 to August 2023), and the third on lung cancer (from March 2023 to August 2024). These CBPR-guided initiatives are designed, implemented, and evaluated in collaboration with community leaders, lay community health workers (CHWs), and other representatives of community members, some of whom were invited to join the Community Advisory Board (CAB). The components address the need and barriers to health information and healthcare services on multiple levels: community-, organizational-, and individual-level. The liver cancer prevention initiative included three components: a community-based, culturally tailored educational campaign; a city-wide awareness campaign through poster advertising in buses; and the distribution of information about HBV, HCV, and liver cancer to community member attendees at community health fairs. This study was determined to be exempt by the Temple University Institutional Review Board (IRB, protocol number 25294). All participants agreed to take part in the program; the requirement for written informed consent was waived by the IRB, as it was deemed unnecessary for this exempt study.

In the following sections, we will detail (1) the engagement of a CAB in various stages of the initiative; (2) CBPR-guided recruitment of participants; (3) the development of culturally tailored education materials; (4) the implementation of the multilevel, multicomponent initiative and its impact on outcomes; and (5) community empowerment and capacity building.

### 2.1. Establishing a Community Advisory Board

CABs often serve as a source of leadership in academic-community partnerships to guide CBPR. They commonly provide a mechanism for community members to have representation in the partnership’s activities (Newman et al. 2011). The COC CAB is composed of 12 influential community leaders from the three targeted populations (4 Hispanic, 4 African American, and 4 Asian American members). Some had long-term collaborative relationships with the COC leaders, whereas others came from organizations that were new partners with missions aligned with the purpose of the SPEECH Partnership and the COC liver cancer prevention initiative. The COC team consistently engages CAB members through quarterly meetings for consultation. CAB has played a critical role in establishing a system-level infrastructure for the SPEECH Partnership’s academic-community initiatives and in promoting institutionalization and sustainability of these initiatives, their products, and their health-related outcomes.

During the development phase of the initiative, CAB served several vital functions. The board members played central roles in (1) reviewing and finalizing the specific objectives of the initiative; (2) providing comments on the content of community educational materials and public awareness campaign messages; (3) consulting on the design and implementation plan of building community capacity and training to CHWs; and (4) advising on the evaluation design (data collection methods and questionnaire content). They participated in regular program planning meetings, where an open and inviting environment was created for honest voices from the diverse communities to be heard. Detailed suggestions from the board members on the interests, needs, values, and concerns of each community regarding liver cancer prevention knowledge were critical in ensuring that the educational materials and awareness messages of this initiative were relevant and culturally sensitive.

### 2.2. CBPR-Guided Participant Recruitment and Data Collection

The engagement of community leaders and stakeholders in participant recruitment was active, direct, and impactful. We first established connections with various community-based organizations (CBOs) in the three targeted populations. These CBOs included churches, temples, adult daycare centers, senior centers, community-service organizations, clubs, barber shops, and other types of community institutions. Community leaders and stakeholders, such as church pastors, healthcare providers, and multilingual community health workers (CHWs), who were familiar with cancer education and had previous collaborative relationships with the program team, were instrumental in outreach to new CBOs to gauge their interest. To establish a common understanding of the shared goals and priorities related to the health needs of the communities, we developed fact sheets on liver cancer and viral hepatitis burden in each community. These fact sheets helped convey the magnitude and urgency of liver cancer prevention to the CBO leaders, thus helping to establish shared goals and priorities between the program team and the CBOs.

In all three racial/ethnic communities, CAB members, collaborating community leaders, and stakeholders served as a liaison between the COC team and the communities, facilitating bi-directional communication on initiative purpose, design, and implementation. CAB members, collaborating community leaders, and stakeholders provided critical input on content design and distribution of recruitment flyers, ensuring that multilingual messages were culturally sensitive and accessible to lay community members. Their role in establishing a shared mission and trust between the research team and the CBOs was crucial to the success of site recruitment. They were also closely engaged in the collection of impact evaluation data. They participated in regular program planning meetings to provide input on survey design to ensure that questions in the survey questionnaire were accessible and relevant.

### 2.3. Development of Culturally Tailored Education Materials

The development of the culturally tailored educational curriculum was detailed in a previous publication (Zhu et al. 2022). We adapted several educational curricula that had proven effective in raising cancer prevention awareness and behavior in minority populations in our previous studies (Ma et al. 2017, 2019). The curriculum included four modules: (1) unique liver cancer disparities in African/Black, Asian, and Hispanic American populations; (2) risk factors of liver cancer; (3) knowledge about HBV/HCV; and (4) healthy eating and liver cancer prevention. For the foundation of the curriculum, we incorporated facts and guidelines on liver cancer and viral hepatitis symptoms, prevention, screening tools, and management from the National Cancer Institute, the Center for Disease Control and Prevention, the Mayo Clinic, and the American Cancer Society.

The cultural tailoring process of the educational materials was guided by the Cultural-Historical Activity Theory (Igira and Gregory 2009). This theory suggests that culture, language, and community are central to learning and stimulating behavior change; it has been widely applied in educational/intervention programs in various settings (Whitney et al. 2017; Andrews et al. 2021). In concert with CAB members and community leaders, we identified important core cultural themes among our targeted communities. Examples include “collectivism”, “religiosity”, “expressiveness”, “intuition”, and “empiricism” for African Americans; “respect for elders”, “familism”, “humility”, “loss of face”, and “respect for authority” for Asian Americans; and “fatalism” and “positive social interactions”, or “simpatia”, for Hispanic Americans (Whitney et al. 2017; Toth-Cohen 2008; Kreuter et al. 2003). The CAB further guided us when conceptualizing cultural tailoring into three core themes: (1) “superficial”, which was related to “languages” and “images” to render materials and programming familiar and appealing to our community and to aid in delivering workshops in native languages; (2) “sociocultural”, which focused on target groups’ cultural values/norms, health belief/stigma, and collective attitude; and (3) “evidential”, which involved presenting pertinent empirical information from authoritative sources to targeted populations. CAB members provided message dissemination guidance. For example, we used bok choy as an example when explaining the amount and types of vegetables in a healthy diet to Asian communities, since the vegetable is familiar to this population. We also added tofu as a source of protein, since it is a food that is commonly consumed in the Asian community. CAB members also recommended incorporating images that better resonate with the real-life experiences of individuals within specific racial or ethnic communities.

### 2.4. Implementation and Impact Evaluation of the Multilevel, Multicomponent Initiative

#### 2.4.1. Initiative Component 1: Educational Workshops

The tailored educational curriculum was delivered to the community in the form of group educational workshops from 2019 to 2021. Individuals needed to be at least 18 years old to participate in the workshops. All workshops were facilitated jointly by multilingual program team members, trained bilingual CHWs, and community leaders and stakeholders at CBOs in the African/Black, Asian, and Hispanic American populations in the PNN region. All workshops before the COVID-19 outbreak (March 2020) were conducted in person at the CBOs, while those after the outbreak were conducted via Zoom or a hybrid approach. To assess the impact of this educational workshop on community members’ knowledge and awareness of viral hepatitis/liver cancer prevention, as well as their preventive behaviors, we collected data through face-to-face, telephone, or online surveys at three time points: pre-workshop, post-workshop, and a 6-month follow-up survey. In total, 578 participants completed the pre-workshop survey, and 541 completed the post-workshop survey. Largely due to the disruptions brought by the COVID-19 pandemic, we were only able to reach 344 participants in the 6-month follow-up survey. Survey data showed that the knowledge score increased significantly from baseline to 9 post-workshop assessment in the total study sample and in each racial/ethnic group. We also found moderately positive changes in dietary behaviors among participants, with distinct racial/ethnic disparities (Zhu et al. 2022).

#### 2.4.2. Initiative Component 2: A City-Wide Awareness Campaign Through Poster Advertising in Buses

From 2019 to 2020, the COC team conducted a city-wide bus campaign in Philadelphia to promote HBV and HCV screening. The campaign featured an educational advertisement on Southeastern Pennsylvania Transportation Authority (SEPTA) buses in Philadelphia. The central message of the ad is, “Liver cancer is on the rise in Philadelphia; Get screened for HBV and HCV”. The program team identified 26 bus routes with 115 buses that travel through areas heavily populated with target populations. The ad posters were placed on the board behind the driver’s seat in buses, presented to an average of 33,510 daily commuters on the bus routes. The ads were placed for four weeks, which meant that about 938,280 riders had a chance to view the ad.

We collected evaluation data from bus riders at the bus stops, based on recommendations from CAB members. In total, 173 passengers responded to the survey. Among them, 22.7% reported seeing the ad. In addition, riders who saw the ad were significantly more likely to report that they had been tested for HBV or HCV (86.5%) than those who did not see the ad (58.3%). This suggested a potential link between HBV/HCV awareness and screening uptake, further highlighting the need for efforts to raise awareness and increase knowledge of the role of HBV/HCV in liver cancer.

#### 2.4.3. Initiative Component 3: Community Health Fairs

Community health fairs are a common way for local government agencies, healthcare organizations and providers, and other stakeholders to perform outreach functions (Mayer et al. 2003). They typically provide an accessible and family-friendly environment for community-dwelling individuals to access health screenings, referrals, and other services and to socialize with friends and other families (Mayer et al. 2003). Health fairs offer unique opportunities for health promotion efforts and campaigns to conduct community outreach and engagement. Because such fairs usually take place in venues where there is no requirement for English proficiency, there is opportunity to provide accurate information about health problems and to promote health actions or behavioral changes to community members for whom English is a second language (Ezeonwu and Berkowitz 2014). Community health fairs are also an opportunity for trust building between academic researchers, healthcare providers, CBOs, and community members, with these parties engaging in dialogue networking, and relationship building. Such activities enable these various groups to become more unified and to provide more coordinated responses to community health issues than what could be achieved by any single party (Cargo and Mercer 2008).

Cultural festivals, community gatherings, and supermarket settings are effective opportunities for reaching community members (Derrett 2003; Getz and Frisby 1988). The COC team utilized these channels, organizing or co-organizing 98 community health fair events and distributing 6056 educational handouts on liver cancer and other cancer types to community members. In addition to the dissemination of information, the COC team coordinated with several health providers and non-profit health organizations to provide health checkups (such as blood pressure tests), vaccine counseling, and COVID vaccine administration to the willing participants. Survey data revealed a suboptimal level of screening for HBV or HCV, with only 28% and 2% of participants, respectively, reporting having been tested for the viruses. After talking with the COC team staff, 46% of participants indicated that they would talk to their doctor about testing in the future. These findings indicated an ongoing need for liver cancer and viral hepatitis awareness campaigns to raise awareness and participation in HBV and HCV screening for liver cancer prevention.

## 3. Results

### 3.1. Evaluation Data: Collection, Analysis, and Co-Ownership

CAB members were closely involved in the development of data collection plans and in the actual collection of survey data. Specifically, they provided input on the accuracy and appropriateness of content translation into multiple languages, survey length, and bus advertisement and health fair brochure content. Furthermore, CAB members provided feedback on the data analysis plans and the interpretation of the evaluation results to make sure the findings would not lead to the re-stigmatization of health behaviors and outcomes in the minoritized populations.

Data co-ownership is a core element of any successful and sustainable relationship between academic institutions and community partners. Under our initiative, we have established a data inventory and a codebook, both of which were shared with CBO leaders, staff, and regular members. A protocol for the application to access data was established for individuals from academic institutions and community organizations to utilize the evaluation data collected. So far, several graduate students and CBO staff members have used the evaluation datasets for their coursework or Master’s-level Capstone projects. CBOs have also used the evaluation findings for program evaluation and annual reports. In addition, ongoing training topics, such as data collection, study design, survey data collection, health promotion, and communication, are provided to community organizations. These steps are essential in achieving community empowerment and in fostering trust and sustainability within the academic-community partnership (Wolff and Maurana 2001).

### 3.2. Community Empowerment & Capacity Building

This initiative created intentional spaces and opportunities for community empowerment in several domains. First, the Initiative allocated small grants to each of 10 CBO partners (two CBOs serving Asian American communities, three serving African American communities, and five serving Hispanic communities) to support their work on this initiative and in other efforts related to cancer prevention and early diagnosis. The selection of these 10 CBOs as grant recipients was based on their demonstrated expertise and prior experience in health promotion.

In addition, evidence gained from post-initiative interviews with CBO leaders and CAB meeting feedback showed that through the active engagement of and training provided to CBO leaders, CHWs, and other stakeholders in the design, implementation, and evaluation of the initiative, the communities were able to enhance their capabilities in event organization, logistics coordination, program management, and progress and outcome monitoring and were able to improve teamwork and communication skills. This increased proficiency empowered the CBOs and their members to confidently initiate and conduct additional campaigns focused on health promotion (on topics such as other types of cancer, diabetes, or mental illness), social justice issues, and social welfare. They were also better prepared to address the various needs of their communities.

This initiative also provided diverse forms of support to communities in their response to and continued recovery from the challenges posed by the COVID-19 pandemic. Thoughtfully incorporating COVID-related information into our liver cancer prevention education enabled us to address the immediate needs of the community without deviating from the original focus of this initiative. To this end, in addition to our efforts on communicating liver cancer-related information, we translated and disseminated information on COVID-19 symptoms, transmission, prevention, testing locations, and treatment resources. Support services also had a role in helping community members overcome emotional trauma caused by anti-Asian racism and discrimination exacerbated by the pandemic. Such support was critical in empowering the communities in mobilizing community members and resources, facilitating recovery from job losses and financial strains caused by the pandemic, and ultimately providing a foundation for community members to shift their attention back to cancer prevention.

## 4. Discussion

This study demonstrated how CBPR principles could effectively guide cancer prevention initiatives in underserved communities through trust building, community empowerment, and community capacity building.

Establishing and building trust is not only the first and most critical step in the beginning of a community–academic partnership, but also essential for the sustainability of the partnership (Wolff and Maurana 2001). Trust building and respect fostering is a long, slow, and oftentimes laborious process that requires patience, passion, and commitment, especially when working with communities that have been minoritized, marginalized, and historically suppressed. In addition, sharing vision, priority, and power is necessary in establishing successful and sustainable relationships between academic institutions and community-based organizations. Relationship building further requires recognition of community inputs and ownership of community-based initiatives.

Through the engagement of the CAB and 51 CBOs, more than 500 community members participated in liver cancer educational workshops and the city-wide campaign survey. The work of the CAB and CBOs also established a foundation for continued community empowerment efforts in cancer prevention and early diagnosis and cancer research participation. In the years since the liver cancer initiative, all participating sites have continued to be actively involved in similar initiatives that are focused on colorectal cancer and lung cancer prevention and early diagnosis.

Training lay CHWs, community leaders, and CBO staff members provided a unique opportunity for community capacity building and community need assessment. Community leaders, lay health workers, and site staff members received training on liver cancer-related facts, communication skills, and health promotion skills. Such training strengthened and honed their capacity to act as community leaders and health ambassadors, assisting them in their efforts to improve community resiliency and health equity. Feedback on the successes and challenges they encountered in the field provided important information on unmet needs, common misconceptions, and barriers to healthcare for community members.

A key methodological contribution of this initiative lies in its establishment of a multi-racial CAB comprising African American, Asian American, and Hispanic community leaders. This structure enables real-time cross-learning and collaboration across racial and cultural groups, fostering cultural awareness and culturally sensitive cancer prevention strategies. The simultaneous engagement of these communities created shared spaces for dialogue and co-creation, contributing to an integrated participatory network.

This initiative is limited by the lack of a control group, hence our inability to determine the degree to which each component may have contributed to changes in participants’ knowledge, attitudes, and behaviors related to liver cancer prevention, or other external factors other than the CBPR-guided workshops and campaign. Furthermore, the outbreak of the COVID-19 pandemic in March 2020 brought notable disruption to the implementation of the program and, in a larger context, to the lives of community members. Racial/ethnic minority communities were particularly hard hit by the pandemic and its economic, social, and emotional consequences. The COC team and CBO partners collaboratively addressed the COVID-19-related challenges. Specifically, in response to public health measures, this initiative promptly switched the educational workshops to a virtual format, mostly via Zoom. In addition, informational and logistical support to communities was incorporated to facilitate the dissemination of accurate medical and public health information on COVID-19, to aid in patient navigation to healthcare access, and to organize a COVID-19 vaccine mobile unit to low-income communities in Northeast Philadelphia. The joint response to the most urgent needs in communities, with coordinated efforts to strengthen community resilience when facing major challenges, was a testimony to the strength of the academic–community partnership.

Despite limitations, this initiative demonstrates that a multi-racial, CBPR-guided academic–community partnership can inform and strengthen sustainable liver disease screening and cancer prevention programs in underserved communities. Establishing a multi-racial CAB within screening initiatives can foster trust, cross-community learning, and culturally relevant outreach. The academic–community partnerships established through this initiative, grounded in shared power, mutual respect, and trust, have laid a strong foundation for sustainable community engagement, continued capacity building, and long-term empowerment in addressing cancer prevention and health disparities. Overall, this CBPR-guided, trust-driven model offers a replicable and effective framework for embedding community voices into health screening and cancer prevention infrastructures, ultimately advancing health and long-term community resilience.

## Data Availability

The data presented in this study are available on request from the corresponding author.

## Abbreviations

The following abbreviations are used in this manuscript:

CAB: Community advisory board
CBO: Community-based organizations
CBPR: Community-based participatory research
CHW: Community health workers
COC: Community Outreach Core
HBV: Hepatitis B virus
HC: Hunter College
HCV: Hepatitis C virus
PNN: Pennsylvania-New Jersey-New York City
SPEECH: Synergistic Partnership for Enhancing Excellence in Cancer Health
TUFCCC: Temple University/Fox Chase Cancer Center
